# MMP-2 and MMP-9 Activities and TIMP-1 and TIMP-2 Expression in the Prostatic Tissue of Two Ethanol-Preferring Rat Models

**DOI:** 10.1155/2015/954548

**Published:** 2015-07-15

**Authors:** Beatriz Aparecida Fioruci-Fontanelli, Luiz Gustavo A. Chuffa, Leonardo O. Mendes, Patricia Fernanda F. Pinheiro, Flávia Karina Delella, Cilmery S. Kurokawa, Sérgio Luis Felisbino, Francisco Eduardo Martinez

**Affiliations:** ^1^Department of Anatomy, Institute of Biosciences, Universidade Estadual Paulista (UNESP), 18618-970 Botucatu, SP, Brazil; ^2^Structural and Cell Biology Program, UNICAMP, 13083-862 Campinas, SP, Brazil; ^3^Department of Morphology, Institute of Biosciences, Universidade Estadual Paulista (UNESP), 18618-970 Botucatu, SP, Brazil; ^4^Department of Pediatrics, Faculty of Medicine, Universidade Estadual Paulista (UNESP), 18618-970 Botucatu, SP, Brazil

## Abstract

We investigated whether chronic ethanol intake is capable of altering the MMP-2 and MMP-9 activities and TIMP-2 and TIMP-1 expression in the dorsal and lateral prostatic lobes of low (UChA) and high (UChB) ethanol-preferring rats. MMP-2 and MMP-9 activities and TIMP-1 and TIMP-2 expression were significantly reduced in the lateral prostatic lobe of the ethanol drinking animals. Dorsal prostatic lobe was less affected showing no significant alterations in these proteins, except for a reduction in the TIMP-1 expression in UChA rats. These important findings demonstrate that chronic ethanol intake impairs the physiological balance of the prostate extracellular matrix turnover, through downregulation of MMPs, which may contribute to the development of prostatic diseases. Furthermore, since these proteins are also components of prostate secretion, the negative impact of chronic ethanol intake on fertility may also involve reduction of MMPs and TIMPs in the seminal fluid.

## 1. Introduction

The ethanol alters the epithelial cells [1], the normal stromal-epithelial homeostasis [2], the inflammation [3], and the concentration of retinoic acid [4] in the prostate. This gland produces and secretes collagenase-like peptidase and gelatinolytic proteinases, such as matrix metalloproteinase (MMP), that are important for reproduction [5–7] and for turnover of the extracellular matrix components (ECM) as collagens, elastins, gelatin, matrix glycoproteins, and proteoglycan [8–10]. MMP-2 and MMP-9 (gelatinases A and B, resp.) are known to play key roles in tissue remodeling and repair through the degradation of many matrix proteins. The activity of MMP-2 and MMP-9 is regulated, respectively, by tissue inhibitors of metalloproteinases termed TIMP-2 and TIMP-1 [11].

The imbalance in MMPs/TIMPs ratio is involved in the development of diseases such as arthritis, cancer cell invasion, metastasis, and fibrosis [8, 12]. Ethanol exposure alters this balance and leads to the development of fibrosis in the liver [13] with an excessive deposition of ECM [14]. In addition, ethanol has also been demonstrated to alter MMP-2 and MMP-9 activities in isolated vascular cells and breast cancer cells [15, 16].

Although it is known that the change of MMPs activities and TIMPs expression may result in pathologic alterations and that ethanol modifies the MMPs activity and TIMPs, the MMP-2 and MMP-9 activities in the prostate, as well as levels of TIMP-1 and TIMP-2 during chronic ethanol intake, have not yet been examined. Such analyses may reveal another facet of the prostatic damage caused by ethanol. Therefore, we evaluated whether chronic ethanol intake alters the MMP-2 and MMP-9 activities and TIMP-2 and TIMP-1 expression in the dorsal and lateral prostates of UCh rats.

## 2. Material and Methods

### 2.1. Animals, Experimental Groups, and Diet

Forty UCh (University of Chile) male rats were used for this experiment. The UCh varieties are ethanol-preferring rats that display voluntary ethanol intake [17]. There are two UCh rat varieties, UChA and UChB, low (inbreeding of rats drinking less 2 g ethanol per kg of body weight/day) and high ethanol consumers (inbreeding of rats drinking more than 2 g ethanol per kg of body weight per day) of 10% (v/v) ethanol solution, respectively [18]. Twenty adults of each variety, UChA and UChB, weighing between 280 and 330 g (~90 days old), were obtained from the Department of Anatomy, Bioscience Institute, Campus of Botucatu, Universidade Estadual Paulista (IBB/UNESP). The UChA and UChB rats were divided into two subgroups (*n* = 10/group): UChA (ethanol-consuming rats) and UChAC (water-consuming rats); UChB (ethanol-consuming rats) and UChBC (water-consuming rats).

The ethanol intake was measured weekly throughout the experimental period (120 days) using a marked test tube. All rats were housed in individual cages in a temperature- and humidity-controlled room under a 12 h light-dark cycle and had free access to filtered tap water and were fed with standard rodent chow Nuvital (Nuvilab CR-1). After exposure, the rats were anesthetized with an intramuscular injection of a 2 : 1 solution of ketamine hydrochloride at 50 mg/mL and xylazine hydrochloride at 20 mg/mL at a dose of (0.1 mL/100 g) and euthanized by exsanguination. The dorsal and lateral prostatic lobes were collected, quickly frozen in liquid nitrogen, and stored at −80°C until the analysis would be carried out. The experimental protocols followed the Ethical Principles in Animal Research adopted by the Brazilian College of Animal Experimentation and were approved by Ethical Committee of the IBB/UNESP (Protocol number 340/2011).

### 2.2. Assessment of Gelatinolytic Activity of the MMP-2 and MMP-9

The gelatin-zymography assay was accessed according to the method described previously by Carvalho et al. [19]. The gelatinolytic activities of MMP-2 and MMP-9 were measured in the dorsal and lateral prostatic tissues from four different rats of each experimental group. The frozen samples were mechanically homogenized in lysis buffer. Then, 35 *μ*g of protein was loaded in SDS-PAGE gels (8%) under nonreducing conditions, containing gelatin at a concentration of 1 mg/mL. Furthermore, purified MMP-2 (20 ng) and MMP-9 (30 pg) (Calbiochem, Boston, MA, USA) were also loaded as positive controls, and molecular weight determinations of MMP-2/MMP-9 were estimated with reference to protein standards (Bio-Rad Laboratories, Inc., Richmond, CA, USA).

Finally, quantitative evaluation of the gelatinolytic activity of captured images of the gels was performed by quantifying the lysis bands corresponding to each type of enzyme activity using a computer-based imaging program (NIH freeware Image-J). The values were expressed as the mean ± SD of the totality of IODs for the MMP-2 proenzyme, intermediate, and active forms and for MMP-9 pro- and active forms.

### 2.3. Measurement of TIMP-1 and TIMP-2

The prostatic tissue expression of TIMP-1 and TIMP-2 was determined using the commercially available immunoassay kit (Quantikine Rat TIMP-1 and TIMP-2) according to the manufacturer's instructions (R&D Systems, Inc.). Prostatic tissue samples were homogenized and diluted 1 : 200 (v/v) for TIMP-1 and 1 : 10 (v/v) for TIMP-2. The concentrations of TIMPs in the samples were determined by extrapolation from an adapted standard curve. TIMPs were determined in the following ranges: TIMP-1 between 37.5 and 2400 pg/mL and TIMP-2 between 1.56 and 100 ng/mL. All determinations were performed in duplicate. The coefficient of variation intra-assay was <4% for TIMP-1 and <10% for TIMP-2. The interassay value was <8% for TIMP-1 and <12% for TIMP-2.

### 2.4. Statistical Analysis

The gelatinolytic activity of the MMP-2 and MMP-9 was evaluated using Student's *t*-test. The analysis of TIMP-1 and TIMP-2 was compared using the Mann-Whitney test. Significant differences were set at *P* < 0.05. The statistical software used was Graph Pad InStat version 3 (Graph Pad Software, San Diego, CA, USA), and Sigma Plot version 11 (Systat Software, San Jose, CA) was used for graphic design.

## 3. Results

### 3.1. Ethanol Intake

The relative ingestion of ethanol by rats was analyzed during the whole experimental period, and the results were as expected for each variety. The daily average of ethanol ingestion by UChA and UChB was 1.7 ± 0.1 and 4.2 ± 0.3 (mean ± SD) g/kg, respectively.

### 3.2. Effects of Ethanol upon MMP-2 and MMP-9 Activity in the Dorsal and Lateral Prostatic Lobes

**Figure 1:**
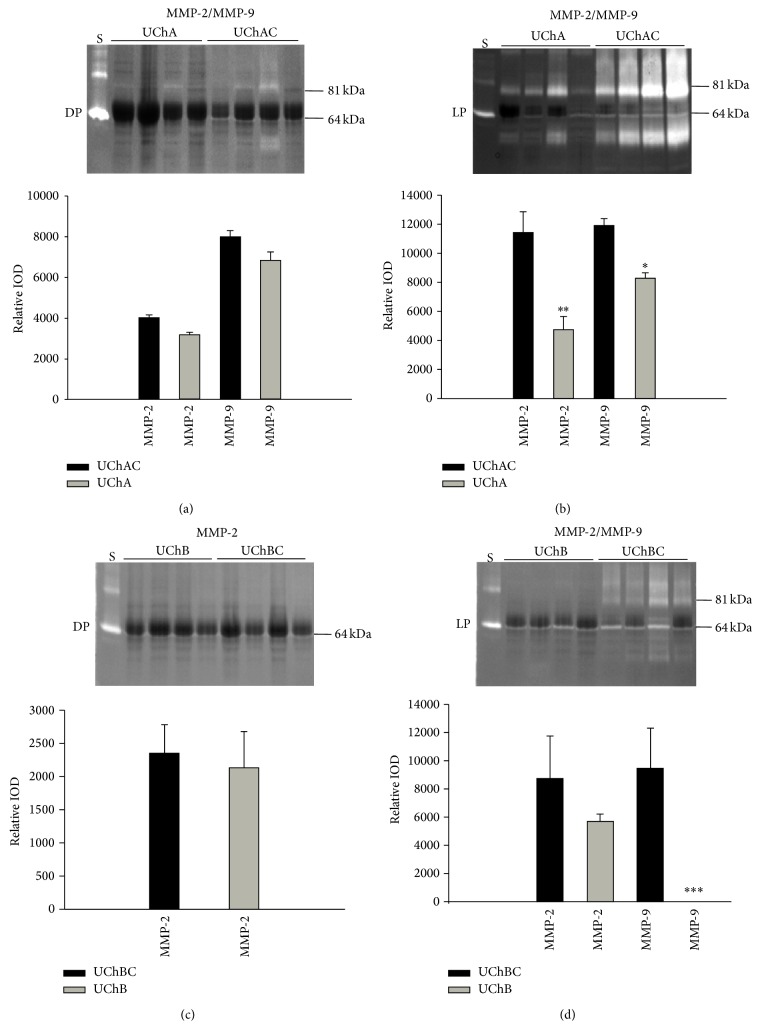
Representative gelatin-zymography of dorsal prostate (DP) and lateral prostate (LP) from low (UChA) and high (UChB) ethanol-preferring rats. UChAC and UChBC are respective controls. Clear bands of gelatinolytic activity for MMP-2 (64 kDa, intermediate form) and MMP-9 (81 kDa, active form) were observed. Lane S corresponds to the reference standard of human pro-MMP9 and active-MMP2 enzymes. Ethanol intake slightly reduces the MMP-2 activity in the dorsal lobe of both rat models and in the lateral lobe of UChB rats but significantly reduces it in the lateral lobe of UChA rats. MMP-9 activity was slightly reduced in dorsal lobe but was significantly reduced in the lateral lobe of both rat models, mainly in the UChB rats. The histograms represent the values of densitometric analysis of the bands. Data are expressed as the mean ± SD (^*∗*^0.01 < *P* < 0.05; ^*∗∗*^0.001 < *P* < 0.01; ^*∗∗∗*^ *P* < 0.001).

The gelatin-zymography assay showed that when compared with control group, MMP-2 activity was slightly decreased in the dorsal prostate of UChA rats (Figure 1(a)) and UChB (Figure 1(c)). In the lateral prostate, MMP-2 activity was significantly decreased in UChA rats (Figure 1(b)) and slightly reduced in the UChB rats (Figure 1(d)). MMP-9 activity was slightly decreased in the dorsal prostate of UChA rats (Figure 1(a)) and was not observed in the dorsal prostate of UChB (Figure 1(c)). In the lateral prostate, MMP-9 activity exhibited a significant reduction in UChA rats (Figure 1(b)) and a marked reduction in the UChB rats (Figure 1(d)).

### 3.3. TIMP-1 and TIMP-2 Protein Expression in the Dorsal and Lateral Prostates

**Figure 2:**
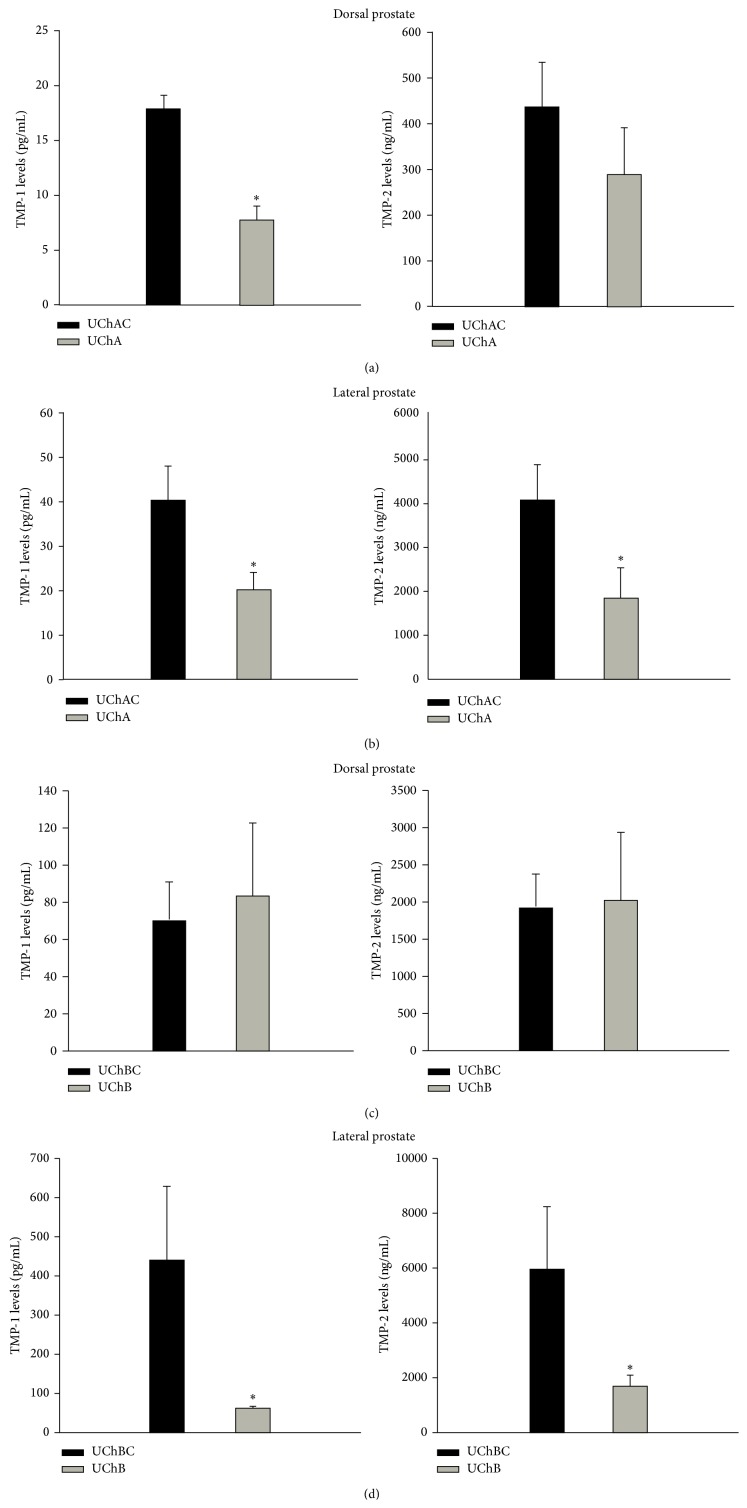
TIMP-1 and TIMP-2 protein levels in the dorsal prostate (DP) and lateral prostate (LP) from low (UChA) and high (UChB) ethanol-preferring rats. UChAC and UChBC are respective controls. The TIMP-1 expression decreased significantly in the dorsal and lateral prostate of UChA rats (Figures 2(a) and 2(b)), and in the lateral prostate of UChB rats (Figure 2(d)). Conversely, TIMP-2 expression was significantly decreased in the lateral prostate of both UChA and UChB rats (Figures 2(a)–2(d)) and slightly reduced in the dorsal prostate of UChA rats. The histograms represent the values of densitometric analysis of the bands. Data are expressed as the mean ± SD. (a) TIMP-1 (*P* < 0.01). (b) and (d) TIMP-1 and TIMP-2 (*P* < 0.05).

The TIMP-1 expression decreased significantly in the dorsal and lateral prostate of UChA rats (Figures 2(a) and 2(b)), and in the lateral prostate of UChB rats (Figure 2(d)). Conversely, TIMP-2 expression was significantly decreased in the lateral prostate of both UChA and UChB rats (Figures 2(a)–2(d)) and slightly reduced in the dorsal prostate of UChA rats.

## 4. Discussion

The amount of ethanol consumed by the UChA and UChB rats allowed us to evaluate their effects upon MMP-2 and MMP-9 activities as well as TIMP-1 and TIMP-2 expression in the dorsal and lateral prostatic lobes. Taken together our results have shown that low (UChA) and high (UChB) chronic ethanol intake decreased the MMP-2 and MMP9 activities in the rat lateral prostatic lobe. The moderate to regular intake of ethanol reduces growth of atherosclerotic plaque by reducing MMP-2 and MMP-9 activities, which are responsible for degrading types IV and V collagen surrounding smooth muscle cell [16]. The moderate ethanol intake is important to prevent vascular diseases in humans [20]. Disruption of the MMP/TIMP ratio can lead to the excessive accumulation of collagen in the extracellular matrix and consequently result in fibrosis [13, 21]. The decreases of MMPs activities observed in the lateral prostate of rats exposed to ethanol can lead to prostatic fibrosis that occurs when the matrix synthesis rate exceeds the degradation [21–23]. Thus, the proteins associated with tissue fibrosis need to be further investigated in the lateral prostatic lobe of ethanol-preferring rats.

TIMPs act as a negative regulator for MMP-9 and MMP-2, and when TIMPs increase, the MMPs activity decreases. There is evidence that TIMPs, mainly TIMP-2, is a bifunctional protein that acts as an inhibitor of MMPs and also as an activator of pro-MMPs [24]. Since both MMPs and TIMPs showed the same direction of reduction of activity/expression, respectively, we did not find an imbalance in the MMP/TIMP ratio regarding the prostatic lobes of ethanol-preferring rats. Low MMPs activities were followed by low TIMPs levels, indicating the expected normal regulation, mainly in the lateral prostate. In the dorsal prostate, the slight decrease in the activity of MMP-2 was correlated with a slight decrease in TIMP-2 levels, while normal activity of MMP-9 was associated to a decrease in the TIMP-1 levels. How ethanol intake is affecting MMPs and TIMPs expression in prostatic lobes remains to be determined and certainly involves complex transcriptional and translational events, activation of the proenzymes, and inhibition of the activated proteinases by other inhibitors [25, 26]. Furthermore, the reduced MMP activities in lateral lobe of ethanol-preferring rats, besides effects on ECM turnover, may also have implication on regulation of apoptosis and cell growth in this lobe [27, 28].

Previous studies have shown that MMP-2 activity displayed significantly different values related to low and high ethanol exposure [15], and high concentration of ethanol (20% v/v) has also been found to not change MMP-2 activity in the rat mesenteric arterial bed [29] or in the alcoholic liver tissue [30]. Notably, our results further revealed a similar inverse dose response relation between ethanol intake and MMP-2 activity in the lateral prostate.

We conclude that chronic ethanol intake impairs the physiological balance of the prostate extracellular matrix turnover, through downregulation of MMPs, which may contribute to the development of prostatic diseases. Furthermore, since these proteins are also components of prostate secretion, the negative impact of chronic ethanol intake on fertility may also involve reduction of MMPs and TIMPs in the seminal fluid.
